# Finger-Floor Distance Is Not a Valid Parameter for the Assessment of Lumbar Mobility

**DOI:** 10.3390/diagnostics13040638

**Published:** 2023-02-08

**Authors:** Luis Becker, Friederike Schömig, Lea Marie-Sophie Cordes, Georg N. Duda, Matthias Pumberger, Hendrik Schmidt

**Affiliations:** 1 Center for Musculoskeletal Surgery, Charité-Universitätsmedizin Berlin, Charitéplatz 1, 10117 Berlin, Germany; 2 Berlin Institute of Health, Julius Wolff Institute for Biomechanics and Musculoskeletal Regeneration, Charité-Universitätsmedizin Berlin, Augustenburger Pl. 1, 13353 Berlin, Germany

**Keywords:** clinical examination, fingertip-to-floor distance, lbp, lumbar flexion, lumbar mobility, spinal mobility

## Abstract

Low back pain (LBP) could be associated with a reduced lumbar mobility. For the evaluation of lumbar flexibility, parameters such as finger-floor distance (FFD) are historically established. However, the extent of the correlation of FFD to lumbar flexibility or other involved joint kinematics such as pelvic motion, as well as the influence of LBP, is not yet known. We conducted a prospective cross-sectional observation study with 523 participants included (167 with LBP > 12 weeks, 356 asymptomatic). LBP-participants were matched for sex, age, height, and body-mass-index with an asymptomatic control cohort, resulting in two cohorts with 120 participants each. The FFD in maximal trunk flexion was measured. The Epionics-SPINE measurement-system was used to evaluate the pelvic and lumbar Range-of-Flexion (RoF), and the correlation of FFD to pelvic- and lumbar-RoF was evaluated. In an asymptomatic sub-cohort of 12 participants, we examined the individual correlation of FFD to pelvic- and lumbar-RoF under gradual trunk flexion. Participants with LBP showed a significantly reduced pelvic-RoF (*p* < 0.001) and lumbar-RoF (*p* < 0.001) as well as an increased FFD (*p* < 0.001) compared to the asymptomatic control cohort. Asymptomatic participants exhibited a weak correlation of FFD to pelvic-RoF and lumbar-RoF (r < 0.500). LBP patients revealed a moderate correlation of FFD to pelvic-RoF (male: *p* < 0.001, r = −0.653, female: *p* < 0.001, r = −0.649) and sex-dependent to lumbar-RoF (male: *p* < 0.001, r = −0.604, female: *p* = 0.012, r = −0.256). In the sub-cohort of 12 participants, gradual trunk flexion showed a strong correlation of FFD to pelvic-RoF (*p* < 0.001, r = −0.895) but a moderate correlation to lumbar-RoF (*p* < 0.001, r = −0.602). The differences in FFD in an individual patient, assuming consistent hip function, may be attributed partially to the differences in lumbar flexibility. However, the absolute values of FFD do not qualify as a measure for lumbar mobility. Rather, using validated non-invasive measurement devices should be considered.

## 1. Introduction

Low back pain (LBP) is a global burden to society with a lifetime prevalence of up to 80 %, leading to a high rate of work absence, loss of productivity, and hospital admissions, resulting in tremendous direct and indirect costs for societies’ healthcare systems and economies [1]. LBP could be accompanied by functional impairment [2,3,4], which is assessed by various outcome parameters. Hereby self-reported outcome measures are used to evaluate different dimensions such as function by the Oswestry Disability Index or the Roland-Morris Disability Questionnaire, the health-related quality of life by Short Form-36, or a pain assessment by different pain scales such as the Numerical rating scale or the Visual-analog scale [5,6,7]. However, LBP can not only be associated with reduced outcome in patient reported outcome measures, but also with detectable decrease in functional testing [6,8,9,10,11]. Therefore, a widespread heterogeneity of tasks for the functional assessment of LBP exists, and different findings might be associated with LBP such as a reduced movement velocity [8], changes in more complex motion sequences with increased flexion-relaxation time, or the most commonly used tool, the range-of-motion [6,9,10,11]. Under conservative treatment, an improvement of pain and function could be observed [3,9]. Therefore, a reproducible and undemanding detection of functional impairment for therapy planning and restoration of spinal function is required [12]. Accordingly, national guidelines for the management of patients with LBP include a clinical examination of the spine with motion analysis to detect functional impairments in spinal mobility [13].

In most cases, the evaluation of lumbar mobility is performed by non-radiological methods. Here, the finger-floor distance (FFD) in full trunk flexion has been established among others as a parameter for the examination of spinal mobility. The FFD is simple to assess, and it has a high responsiveness and a high reliability [14,15]. Additionally, the FFD has a high correlation to self-assessed impairment due to LBP measured by the Roland Morris Disability Questionnaire [15].

However, the extent to which lumbar and hip mobility correlate with the FFD is currently unknown. Considering that LBP influences the movement distribution of lumbar and hip flexion in forward bending [16], the aim of this study is to assess the validity of FFD as a parameter to represent both, lumbar and hip mobility, in participants with LBP over those without.

## 2. Materials and Methods

### 2.1. Study Design

We performed a prospective cross-sectional observation-study approved by the local ethics board (EA4/011/10). Participants gave written informed consent. The study was reported according to the STROBE guidelines.

### 2.2. Participants

We included 620 participants aged ≥18, with chronic LBP ≥ 12 weeks or without LBP. Chronic LBP was assessed with a questionnaire asking for the subjective condition of persistent LBP for at least 12 weeks, which was defined as chronic LBP. No minimal threshold for LBP on a pain scale, such as a numeric rating scale, was set. The exclusion criteria were acute LBP < 12 weeks, prior spinal surgery, neurological impairments such as paralysis, muscle weakness, radiculopathy, or movement disorders, malignancy, professional athletes, and pain exacerbation during examination, limiting the subjective performance in the range of motion. Therefore, 97 participants were excluded, resulting in 523 included participants, of which 167 stated to have chronic LBP for ≥12 weeks.

### 2.3. Epionics SPINE Measurement Device

**Figure 1:**
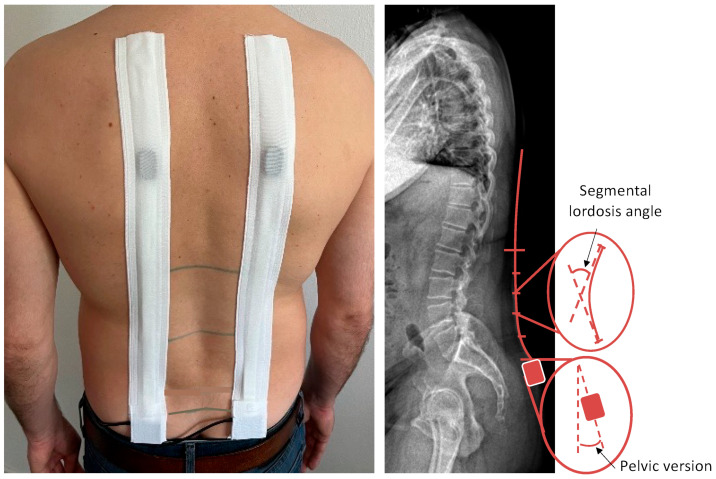
Epionics-SPINE measurement system.

The FFD and pelvic motion (pelvic-RoF), as well as the lumbar Range-of-Flexion (RoF) under maximal trunk-flexion, was observed. For the measurement of pelvic-RoF and lumbar-RoF during trunk flexion, the Epionics-SPINE (Epionics Medical GmbH, Potsdam, Germany) system was used. Epionics-SPINE is a validated, non-invasive measurement device [9,10] that can assess the lumbar back shape and motion via two sensor strips. The system is based on strain-gauge technology, consists of twelve 25-mm long sensor units and a three-dimensional accelerometer at the lower end for the assessment of pelvic version. The two sensor strips are attached to the back 7.5 cm paravertebrally in a standardized manner in hollow plasters. The lower end with the accelerometer is attached at the level of the posterior superior iliac spine. For upper orientation of sensor strips, 15 cm and 25 cm cranially to the spinae iliacae posteriores superiores, markings were attached on both sides 7.5 cm paravertebrally (Figure 1).

To account for spine length discrepancies, the sensor units corresponding to the lumbar lordosis were determined for each study participant, defined by segmental lordosis angle. These sensor units served as reference units for further evaluations for lumbar-RoF. To determine lumbar lordosis in standing and lumbar curvature in maximum flexion, all local angles of the reference sensor units in standing and in maximum flexion were summed individually. The resulting RoF was then calculated as the angular difference from the standing reference. The values of the left and right sensor strips were averaged.

### 2.4. Measurement Protocol

Measurements were performed according to a standardized measurement protocol in all 523 participants. Anthropometrics were measured, body height via stadiometer and body weight via calibrated scale, and the body mass index (BMI) was calculated. This was followed by the application of the Epionics-SPINE measurement device. For standardized assessment of pelvic version and lumbar lordosis in upright standing, participants were asked to step on a platform of 30 cm height with marked foot positions of about 40 cm distance in between and asked to stand upright with fully extended knees. In this position, pelvic version and lumbar lordosis were recorded by the Epionics-SPINE system. Participants were asked to perform maximal trunk flexion with the aim to reach the floor with the fingertips while standing with the knees fully extended. In end position minimal vertical FFD was measured. As participants stood on a platform 30 cm high for measurements, a measured FFD of 30 cm was labeled and reported as 0 cm, referencing to standing on the ground floor, whereas the lowest reachable value for FFD was -30 cm. Pelvic-RoF and lumbar-RoF were assessed by the attached Epionics-SPINE system in end position. Differences of pelvic version and lumbar lordosis between upright standing and trunk flexion end position were calculated and defined as pelvic-RoF and lumbar-RoF.

### 2.5. Measurement Protocol “Gradual Trunk Flexion”

In a randomly chosen sub-group of 12 asymptomatic participants (four females, eight males) without LBP, the proportions of pelvic- and lumbar-RoF during stepwise trunk flexion was assessed. Therefore, a standardized measurement protocol with the same standardized standing and foot position as in the main protocol was established. Firstly, the FFD of participants was measured by measuring tape, and pelvic version and lumbar lordosis in upright standing were recorded. Subsequently, participants were instructed to perform a trunk flexion with extended knees so that the FFD was 60 cm and pelvic-RoF and lumbar-RoF were recorded by the Epionics-SPINE system. After trunk flexion, participants were instructed to return to the relaxed standing position and perform gradual trunk flexion to reach predefined finger-floor distances (60 cm, 52 cm, 44 cm, 36 cm, 28 cm, 19 cm, 10 cm, 0 cm) with intermitting rest periods in the relaxed upright standing position. If participants were unable to achieve a prescribed FFD, no further movement steps were performed.

### 2.6. Data Analysis

Data were tested for normal distribution using the Kolmogorow–Smirnow-test. For comparison of unpaired parametric parameters, the *t*-test, and for continuous data, the Mann–Whitney U-test was performed. For the comparison of two paired parametric samples, the paired *t*-test and for continuous samples, the Wilcoxon-rank sum-test was used. Correlations were observed by Spearman’s correlation coefficient. A *p*-value < 0.05 was considered as statistically significant. Statistical analysis was performed using SPSS Version 27 (IBM Corporation, New York, NY, USA). As other aspects of this cohort were already published elsewhere [17], we performed a post-hoc power analysis with our effect size of 0.442, an α-error of 0.05, and a sample size of 523 participants; a test power of 1.000 was achieved for our cohort. Power analysis was performed using G*Power Version 3.1.9.6.

## 3. Results

### 3.1. Demographics

A total of 523 participants was analyzed. The demographics for the collective of 523 participants and for matched cohorts are given in Table 1.

### 3.2. Sex Differences in Pelvic Version, Lumbar Lordosis, Pelvic- and Lumbar-RoF

The Kolmogorow–Smirnow test showed for the collective of 523 participants the normal distribution for lumbar lordosis and pelvic-RoF, whereas the pelvic version, lumbar-RoF, and FFD did not follow normal distribution. For the whole sample, female and male participants differed significantly in their pelvic version and lumbar lordosis in upright standing (Table 2). The pelvic-RoF, but not the lumbar-RoF, was significantly associated with sex.

### 3.3. Influence of LBP

In the matched cohorts of 120 participants, each pelvic version, lumbar lordosis, pelvic-RoF, and lumbar-RoF followed the normal distribution according to the Kolmogorow–Smirnow test, although the FFD did not follow normal distribution. LBP patients had a significantly reduced pelvic version and lumbar lordosis in upright standing (*p* < 0.001) and significantly reduced pelvic-RoF (*p* < 0.001) and lumbar-RoF (*p* < 0.001) accompanied by a significant increase in FFD (*p* < 0.001) compared to the asymptomatic control-group (Table 3).

### 3.4. Correlation between Lumbar and Pelvic-RoF and FFD

For all study participants, the FFD had a moderate negative correlation with pelvic-RoF (*p* < 0.001, r = −0.548) and a weak negative correlation with lumbar-RoF (*p* < 0.001, r = −0.442). However, individuals with and without chronic LBP differed in the correlations.

**Figure 2:**
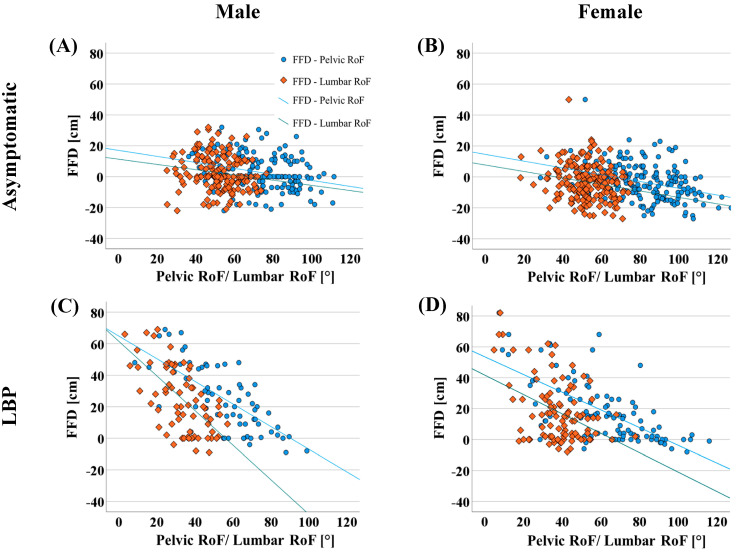
Correlation of lumbar-RoF and pelvic-RoF with finger-floor distance in asymptomatic males (**A**), asymptomatic females (**B**), symptomatic males (**C**), and symptomatic females (**D**).

For the asymptomatic population, a weak negative correlation (*p* < 0.001, r = −0.348) between FFD and pelvic-RoF and no correlation with lumbar-RoF (*p* < 0.001, r = −0.184) was observed. Participants with LBP had a moderate negative correlation between pelvic-RoF and FFD (*p* < 0.001, r = −0.663) and a weak correlation of FFD with lumbar-RoF (*p* < 0.001, r = −0.432). Sex influenced the correlation between FFD and pelvic-RoF and lumbar-RoF. Females and males differed significantly in FFD (*p* < 0.001). In asymptomatic females, pelvic-RoF showed a weak negative (*p* < 0.001, r = −0.353), and in asymptomatic males, it showed a negligible negative correlation (*p* < 0.001, r = −0.295) with FFD. Lumbar-RoF in asymptomatic females (*p* = 0.022, r = −0.163) and males (*p* = 0.012, r = −0.256) also had negligible correlations with FFD. In contrast, for LBP participants, males presented a moderate negative correlation of both pelvic-RoF (*p* < 0.001, r = −0.653) and lumbar-RoF (*p* < 0.001, r = −0.604). However, females showed a moderate negative correlation for pelvic-RoF (*p* < 0.001, r = −0.649) but negligible correlation for lumbar-RoF (*p* = 0.012, r = −0.256) with FFD (Figure 2).

### 3.5. Relationship between Pelvic-RoF, Lumbar-RoF, and Gradual FFD

**Figure 3:**
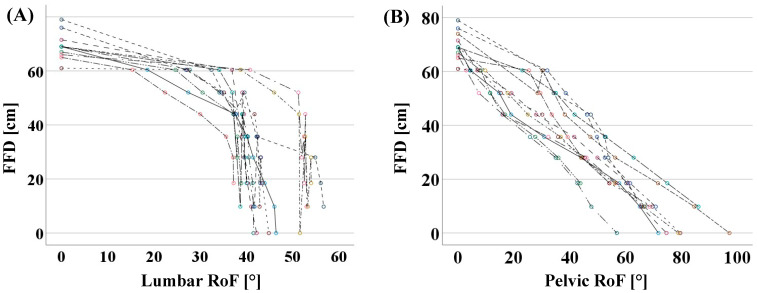
Relationship between gradual FFD and lumbar-RoF (**A**) and pelvic-RoF (**B**) with linear interpolation lines in the subcohort of 12 asymptomatic participants. For each participant the values were depicted in circles of one color.

For the subcohort of 12 asymptomatic participants in which FFD was measured stepwise from 60 cm to 0 cm, four females and eight males with a median (interquartilrange) age of 33.0 (10.8) years, median height of 180.0 (15) cm, and median BMI of 22.1 (10.8) kg/m^2^ were analyzed. The subgroup analyses showed that pelvic-RoF had a strong negative correlation with gradual FFD (*p* < 0.001, r = −0.895), whereas lumbar-RoF yielded a moderate negative correlation with gradual FFD (*p* < 0.001, r = −0.602) (Figure 3).

## 4. Discussion

This study aimed to evaluate the validity of FFD as a measure of lumbar mobility. Our results demonstrate that in a population without LBP, FFD has a weak correlation to both pelvic- and lumbar-RoF. However, in participants with LBP, a moderate correlation of FFD to pelvic-RoF and a sex-dependent correlation to lumbar-RoF was observed. Males with LBP had a moderate correlation of FFD to lumbar-RoF, whereas females presented a weak correlation of FFD to lumbar-RoF. For gradual trunk flexion in 12 individuals, a strong correlation of FFD to pelvis and a moderate to lumbar-RoF was shown.

The obtained values for pelvic-RoF and lumbar-RoF are in the range of those reported elsewhere for maximum trunk flexion [18,19,20,21,22,23]. Consistent with a meta-analysis, we detected sex differences for lumbar lordosis in upright standing with increased lumbar lordosis in females [24]. In agreement, females in our study concomitantly showed an increased pelvic version [18,25,26,27]. Females performed a significantly increased pelvic-RoF during trunk flexion compared to males, due to an increased hip flexion as described in the literature [28]. Lumbar flexion did not differ significantly between sexes [24]. This resulted in a significantly decreased FFD in females compared to males.

Chronic LBP significantly influenced posture. As in our collective, Chun et al. demonstrated an association of LBP with flattening of the lumbar spine [29]. Concomitantly, LBP patients in an upright standing exhibited a significantly reduced pelvic version in line with the results of Schmidt et al. [17]. The association of LBP with restricted RoF is controversially discussed [2,3,30,31]. However, according to a meta-analysis by Laird et al., our results show reduced lumbar mobility in subjects with LBP [2]. Similarly, subjects with LBP presented a significantly reduced pelvic-RoF, which has also previously been described by Wong et al. [16]. The task-related avoidance behavior of subjects with LBP [32] and the reduced hip-spine kinematics with hamstring affection in the context of LBP could possibly have contributed to these findings [33]. The reduced pelvic-RoF and reduced lumbar-RoF resulted in a greater FFD in participants with LBP.

In addition to the outlined differences of posture and RoF between male and female participants, as well as participants with and without LBP, varying correlations between FFD and pelvic-RoF and lumbar-RoF were observed. For asymptomatic participants, a weak correlation was observed for pelvic- and lumbar-RoF. The weak correlation may be due to the complexity of the movement resulting from the numerous movement components such as hip, lumbar, and thoracic flexion, as well as shoulder, elbow, wrist, and finger extension. Perret et al. reported a strong correlation between the FFD and the radiographic evaluation of the tilting of the fifth thoracic vertebra between the standing and trunk flexion [14]. However, these results are comparable with our data only to a limited extent due to the evaluation of a combined movement of hip, lumbar, and thoracic flexion by Perret et al. [14]

The FFD is influenced by anthropometric data such as arm-to-leg length ratio and trunk length. These parameters show a significant correlation with height but a considerable interindividual variability [34], possibly resulting in an influence on the assessment of FFD. This was also observed in our subcohort of 12 participants who were examined with gradual FFD. In these participants, the values of FFD in upright standing differed by as much as 10 cm in individuals of the same height. Accordingly, the individual examination of the FFD versus the gradual trunk flexion, which is resistant against anthropometric differences by patient-specific examination, showed a strong correlation of the FFD to pelvic-RoF, as well as a moderate correlation to lumbar-RoF. As a result, a relevant influence of the body proportions on the FFD could be assumed. Furthermore, the different motion patterns of the trunk flexion in the whole cohort might be too heterogeneous between participants to obtain a strong correlation between the FFD and lumbar-RoF.

However, in participants with LBP, a stronger correlation of FFD to pelvic-RoF was observed. This finding could possibly be attributed to a change in kinematics with a relative reduction in lumbar-RoF in participants with LBP, as described by Wong et al. [16]. In our LBP cohort, males demonstrated a stronger correlation of FFD and lumbar-RoF compared to females. This may result from a greater FFD of males with an LBP of 21.5 cm compared to females with an FFD of 14.0 cm.

In line with our results, the literature shows that in the initial phase of trunk flexion, lumbar-RoF predominates, but in advanced trunk flexion, further hip flexion with concomitant pelvic-RoF occurs almost exclusively [18]. However, these findings have never been considered in relation to FFD. These effects are evident considering the results presented in Figure 3, illustrating that lumbar flexion occurs especially in the initial phase of trunk flexion and, accordingly, the reduction of FFD shows a higher correlation with lumbar flexion, especially in the initial phase of trunk flexion.

This study has limitations that need to be mentioned. Even though this study included a large collective of subjects with and without chronic LBP, a further evaluation of pain intensity based on the available data is not possible, which may also have influenced the presented results. The presumed underlying cause of LBP was not considered, as no imaging data of the participants was available. Therefore, no allocation to specific- or non-specific LBP was performed. Hip–spine interaction could be influenced by hip osteoarthritis, which was not evaluated in our study due to lacking imaging data [35]. In our cohort for patients with a pain increase in trunk flexion, the examination was stopped and the participants were excluded to prevent an elevated FFD caused by pain avoidance behavior. However, this could have resulted in a selection bias and influenced the FFD in the cohort of LBP patients as only participants without subjective pain-related movement restrictions were included. The side deviation and lateral shift in forward bending were not recorded, although the side differences between the left and right side were averaged. Therefore, our results do not take trunk or kinematic asymmetries into account. Even though measuring back shape by Epionics-SPINE is validated against radiographic imaging for the detection of spinal shape [36] differences in soft tissue, the anatomy due to sex or BMI could have influenced our results.

## 5. Conclusions

Based on our results, it could be concluded that the differences in the FFD in an individual patient in longitudinal measurements under consistent hip function may be attributed partially to differences in lumbar flexibility. For the patient-specific examination of stepwise trunk flexion, which is not subject to differences in anthropometrics or to heterogeneity in movement patterns, a strong correlation between FFD and pelvic-RoF and a moderate correlation to lumbar-RoF were found in asymptomatic participants. Based on our results, absolute values of FFD should not be used as a comparative parameter between patients with the purpose of evaluating lumbar flexibility for both asymptomatic and patients with LBP. Instead, the measurement of lumbar RoM by non-invasive measurement devices might be considered a viable option with a high reliability and validity.

## Figures and Tables

**Table 1:** Demographics and anthropometrics of included participants in both the LBP and the control groups.

	Overall				
	LBP (*n* = 167)		Asymptomatic Control (*n* = 356)		
	Male Median (IQR)	Female Median (IQR)	Male Median (IQR)	Female Median (IQR)	
*n*	70	97	159	197	
Age [years]	49 (22)	51 (20)	36 (21)	37 (22)	
Height [cm]	178 (11)	167 (8)	179 (11)	168 (9)	
BMI [kg/m^2^]	26.3 (6.0)	25.8 (6.1)	24.0 (2.0)	22.0 (3.0)	
	**Matched Groups**				
	LBP (*n* = 120)		Asymptomatic Control (*n* = 120)		*p*-value
	Male Median (IQR)	Female Median (IQR)	Male Median (IQR)	Female Median (IQR)	
*n*	49	71	49	71	1.000
Age [years]	49 (23)	50 (22)	48 (18)	49 (22)	0.804
Height [cm]	178 (12)	168 (9)	178 (14)	168 (7)	0.802
BMI [kg/m^2^]	24.9 (3.8)	24.1 (5.1)	25.0 (2.0)	24.0 (4.0)	0.380

IQR = Interquartile range; LBP = low back pain; BMI = body-mass index. Wilcoxon rank-sum test was performed for intergroup comparison between the LBP and the matched asymptomatic control group.

**Table 2:** Sex differences in pelvic version, lumbar lordosis, pelvic- and lumbar-RoF between male and female participants.

	All Median (IQR) (*n* = 523)	Male Median (IQR) (*n* = 229)	Female Median (IQR) (*n* = 294)	*p*-Value
Pelvic version [°]	17.9 (11.7)	16.0 (11.8)	19.0 (10.9)	**<0.001**
Lumbar lordosis [°]	30.8 (14.4)	28.3 (16.0)	32.1 (13.4)	**<0.001**
Pelvic-RoF [°]	72.9 (30.1)	69.5 (28.4)	76.2 (27.7)	**<0.001**
Lumbar-RoF [°]	48.5 (17.8)	48.0 (19.4)	49.5 (16.6)	0.277
FFD [cm]	1.0 (20.0)	5.0 (19.5)	0.0 (20)	**<0.001**

IQR = Interquartile range; FFD = Finger-floor distance; Statistically significant *p*-values are marked in bold.

**Table 3:** Differences in pelvic version and lumbar lordosis, as well as pelvic- and lumbar-RoF, between matched LBP participants and the control group.

	LBP Median (IQR) (*n* = 120)	Control Median (IQR) (*n* = 120)	*p*-Value
Pelvic version [°]	13.5 (11.0)	17.7 (11.9)	**<0.001**
Lumbar lordosis [°]	23.7 (11.9)	30.5 (16.2)	**<0.001**
Pelvic-RoF [°]	61.2 (29.4)	74.2 (26.3)	**<0.001**
Lumbar-RoF [°]	37.7 (15.8)	50.7 (14.6)	**<0.001**
FFD [cm]	16.5 (33.3)	0.0 (15.0)	**<0.001**

IQR = Interquartile range; FFD = Finger-floor distance; Statistically significant *p*-values are marked in bold.

## Data Availability

All data regarding the findngs in the manuscript can be found in the manuscript.
